# Living Close to Nature

**Published:** 1917-01

**Authors:** 


### Living Close to Nature.

The sleeping porch has done much to bring many people back to health. There is hardly a physician now who is not an advocate of the sleeping porch where it is possible to use one, and if not that, then of sleeping in a room that is kept open and well ventilated summer and winter. The old idea that night air was poisonous to anyone has gone with many other of the old false beliefs. People who are sick should not only sleep in the open as much as possible but they should live in the open as much as they can. For that matter, the well should do the same thing to avoid sickness.

When we think of the way people live under our luxurious system of living, there should be no surprise that there is so much sickness. People fill their stomachs with all kinds of highly seasoned and indigestible foods; they attend dinners and banquets and imbibe liquors enough to nauseate a stomach not already liquor diseased, and breathe the smoke that has already gone through the diseased heads of other smokers; and then they wonder that they do not stay well. To stay well one should have good habits, and to get well when one is sick, the best of care should be taken, and the most favorable surroundings should be obtained.

While the sleeping porch is to be commended, invalids would get better results often if they would get out in the open and sleep on the ground or so close to it as to breathe in the moisture of the soil itself. Hunters, fishermen, and others who sleep out in camps, spreading their beds right on the soil seldom become invalids. Why do people long so intensely to go on camping expeditions at certain seasons? It isn't so much for the recreation or the sport, for recreation and sport are not so fascinating to many; it is due to the desire to live by day and night close to the soil, to literally breathe the atmosphere of the land.

The physician who can induce his patients to live in this way while under treatment will assuredly find, in mosts cases, that the life counts for more than the medicine.

